# MAintAin Activity in Arthritis: A study protocol of the co-development and feasibility testing of a physical activity maintenance intervention

**DOI:** 10.12688/hrbopenres.14002.1

**Published:** 2024-12-10

**Authors:** Suzanne McDonough, Aoife Stephenson, Chloe Grimmett, Kathleen Bennett, Noreena Coyle, Stacey Grealis, Claire Kinneavy, Oliver Fitzgerald, Helen French, Maria Stokes, Aisling Walsh, Mick Thacker, Deirdre Hurley Osing, David French

**Affiliations:** 1 School of Physiotherapy, Royal College of Surgeons in Ireland, Dublin, Leinster, Ireland; 2 University of Southampton, Southampton, England, UK; 3 Royal College of Surgeons in Ireland Division of Population Health Sciences, Dublin, Leinster, Ireland; 4 PPI Author, Royal College of Surgeons in Ireland, Dublin, Leinster, Ireland; 5 University College Dublin School of Medicine, Dublin, Leinster, Ireland; 6 University College Dublin School of Public Health Physiotherapy and Sports Science, Dublin, Leinster, Ireland; 7 The University of Manchester Manchester Centre for Health Psychology, Manchester, England, UK

**Keywords:** Physical activity, exercise, arthritis, maintenance, co-design

## Abstract

**Background:**

Despite the wealth of evidence demonstrating the health benefits of physical activity (PA), people with arthritis commonly do not meet recommended PA levels. Whilst various programmes support people with arthritis to become active, most individuals reduce their level of PA after completion of a structured exercise programme. This research aims to co-develop and feasibility test a PA maintenance intervention for those living with arthritis, after exit from a structured exercise programme.

**Methods:**

Intervention development was guided by the INDEX framework for developing complex interventions.

Phase 1, Evidence Synthesis: Bring together existing evidence, clinical guidelines and behavior change theories for PA maintenance in arthritis, to develop an intervention logic model.

Phase 2, Observation and qualitative study: Conduct an observational analysis of a physiotherapy led structured exercise programme for those living with arthritis, to understand what behaviour change components are used and what might support PA maintenance. Followed by a qualitative exploration of PA maintenance barriers, facilitators and strategies for those who have participated in the classes, their family members/friends and the delivering physiotherapist.

Phase 3, Finalise intervention prototype: Results from phases 1 and 2, will be triangulated to inform potential intervention options. Those living with arthritis/key stakeholders will be invited to participatory workshops to refine intervention content and delivery modes.

Phase 4, Feasibility Study: The final phase is a pre-post, mixed methods feasibility evaluation of the newly developed multicomponent PA maintenance intervention for people living with arthritis, after completion of a physiotherapy led structured exercise programme.

**Discussion:**

Intervention development will bring together PA maintenance theory and evidence with user input and other key contextual factors. User input will be achieved by collaboration with two embedded patient researchers and a wider Public Patient Involvement (PPI) panel to ensure diverse patient experiences and perspectives are heard and inform programme design.

## Introduction

There is a wealth of evidence that physical activity (PA) protects health across the life course, especially for those living with chronic health conditions. Despite this, insufficient amounts of PA remain one of the major behavioural challenges worldwide ^1^. Many Irish adults are not meeting the PA guidelines ^2^, and those living with health conditions such arthritis, are even more likely to be inactive. This places them at risk of developing further health conditions and exacerbating their need for rehabilitation ^3^.

### Physical activity and its benefits

Physical activity is defined as any movement produced by skeletal muscles resulting in energy expenditure, and includes all PA done as part of daily living such as social and domestic activities, commuting, recreational and leisure activities ^4– 7^. PA is consistently recommended in arthritis management guidelines with moderate evidence of benefits on cardiovascular fitness, muscle strength, pain management and disability on completion of interventions ^8, 9^. PA interventions also show short-term effectiveness in supporting people to be active ^10^. However, there is no evidence for the long-term maintenance of PA.

### Programmes to support increases in PA

Strategies to support people with arthritis to be active include health service or third sector exercise programmes delivered in person and increasingly virtually, since the COVID-19 pandemic ^10, 11^. Many individuals reduce their level of PA after completion of a prescribed programme ^8, 12, 13^. It is well established that maintenance is critical to sustain clinical benefits of exercise programmes and protect long-term health ^8, 14^. One reason for this decline in activity level may be that interventions designed to support the initiation of PA (e.g. supervised exercise programmes) typically cease without planned contact and follow-up activities. Further, the programmes generally lack the necessary behaviour change components for PA maintenance, such as becoming autonomous and creating new habits ^15^. They typically also neglect to take into account those factors that people find enjoyable or satisfying, such as social contact and enjoyment of activities. These are known to be important for maintenance, particularly in older and more socially isolated people ^16, 17^, and therefore should be taken into account when developing long-term PA maintenance interventions ^17^.

### Physical Activity maintenance

Although there is no consensus on how to define PA maintenance ^18– 21^, both stage-based ^20^ and time-based definitions ^22, 23^ have been used. In stage-based definitions, there is a recognition that behaviours for the initiation of PA differ from those needed for maintenance. PA maintenance is achieved when the behaviour becomes the ‘dominant response’, or an automatic response ^24, 25^. Time based definitions focus on the duration since the behaviour was initiated at a volume that differs from baseline PA e.g. regular activity or statistically significant change in behaviour over one to 12 months ^22, 23, 26^. The duration from behavioural initiation to maintenance varies for time-based definitions e.g. continuing to be active between three to 12 months following the end of an exercise programme ^12^. In previous scoping and systematic reviews, we have included studies with a follow up time point of at least three months after intervention end ^12, 27^.

### Starting to become active

Evidence suggests that for PA interventions to be effective, they should be informed by behaviour change theory which supports people to incorporate PA into their daily life ^28^ (synthesis of 100 reviews). Emerging evidence also suggests that this applies to people living with arthritis. A meta-analysis of 11 randomised controlled trials (RCTs) of counselling-based PA interventions demonstrated small increases in PA levels ^8^. The European League Against Rheumatism (EULAR) 2018 guidelines for the first time, now specifically recommend PA as an integral part of standard management; acting as a bridge between arthritis-specific exercises and public health PA recommendations ^14^.

### Existing maintenance programmes

In arthritis, there is some evidence to support behaviour change approaches delivered digitally ^8, 29, 30^ and non-digitally ^8, 9, 14^ to initiate PA behaviour. A range of booster strategies have been reported such as phone calls, devices, home visits, logbooks, web-based instructions, written material and visual instructions ^8^. However, even with such behaviour change support their effectiveness regresses to previous levels once the intervention is completed ^31^. This means we know very little about how to support people to maintain PA behaviour long-term. Existing literature in this area demonstrates a lack of reporting of maintenance outcomes, small improvements at follow-up that often diminish over time or no significant differences in PA maintenance outcomes ^23, 32^. Another study reported on the feasibility of a maintenance intervention for older adults following a falls programme ^33^. The intervention was feasible and a small increase in PA was identified in both intervention and control groups at 6-months follow-up. The intervention was, however, not designed for people with arthritis and the theoretical underpinning was unclear.

The overall aim of this piece of research is:

To co-develop and feasibility test a PA maintenance intervention for those living with arthritis after exit from a physiotherapy led structured exercise programme. Intervention development will be informed by four study phases that are guided by the MRC framework ^34^, and IdentifyiNg and assessing different approaches to DEveloping compleX interventions framework (the INDEX study) ^35– 37^. 

This aim will be achieved through five objectives, conducted over four study phases

(i) Synthesis of PA maintenance theory and evidence
(ii) Identify facilitators/deficits in supervised exercise classes to maintain PA
(iii) Identify the support needs of people living with arthritis, their family members/friends and the delivering physiotherapists, to maintain PA.
(iv) Co-develop a PA maintenance intervention prototype
(v) Test the feasibility of the PA maintenance intervention prototype

## Phase 1: Objective (i) Synthesis of PA maintenance theory and evidence

We will bring together theories for PA maintenance from key papers ^20, 24, 38– 40^, existing scientific evidence (see below for more detail), best practice recommendations for PA including guidelines from the World Health Organisation ^5^, the UK Chief Medical Officers ^41^, Irish PA guidelines ^42^, best practice recommendations for PA related health behaviour change ^43, 44^ and PA clinical guidelines for arthritis ^14, 45, 46^.

The synthesis will use a programme theory approach to establish an explicit logic model of why the intervention should work, and contextual influences on this ^34^. This approach will allow us to define the theoretical and practical processes and potential mechanisms that lead to PA maintenance. It will also allow for the identification of the commonalities across the arthritis conditions to co-develop the intervention and implementation plans accordingly. For example, differences in PA capability can be addressed through tailoring, while in relation to the opportunities and motivations to maintain PA, there is likely to be overlap in the barriers/facilitators across people with different types of arthritis. We will consider the complexity of the intervention, particularly interactions between proposed components, how the intervention is proposed to fit in its wider context (with stakeholders), and its relevance for people with one or more type of arthritis. 

### Synthesis of the scientific evidence

Our team has published two reviews on digital tools to support PA maintenance ^12, 27^. A third key source is a comprehensive review of PA maintenance literature in older adults ^47^. To add to this information, a rapid review will be conducted to explore the effectiveness of non-digital interventions for PA maintenance in people with arthritis and extract potential intervention components.

### Rapid review

***Methods***

We will use rapid review methodology to accelerate the process of conducting a traditional systematic review through streamlining methods to produce more timely information ^48^. Rapid reviews adhere to the general steps of the systematic review process, use abbreviated methods from full systematic reviews, and are completed within shorter time periods ^48^.

### Data sources and searches

The search process will be accelerated by using a hybrid method to identify studies; this starts by identifying trials and qualitative data from relevant systematic reviews, supplemented by a search for randomised controlled trials (RCTs) published since the last search date of the reviews, if required. We will identify relevant reviews from those already completed by the team ^8, 9, 12, 16, 23, 26, 48^. These reviews will inform the rapid review and will accelerate our search process. The proposed rapid systematic review will follow the updated Preferred Reporting Items for Systematic Reviews and Meta-Analysis reporting guidelines ^49^. Assistance of an information specialist will be obtained to ensure a comprehensive search of electronic databases such as EMBASE, PubMed, CINaHL. Google Scholar will be searched for additional citations associated with final included studies. We will attempt to contact authors of published abstracts to request full-text versions of the study and/or study data. Reference lists of selected studies will be hand searched for additional studies.

Eligibility criteria are outlined using the PICOS framework ^50^:

P (Population) = Adults living with osteoarthritis, RA (plus other forms of arthritis to be agreed)

I (Intervention) =, non-digital PA maintenance intervention (Maintenance defined as at least 3-months after the end of the intervention)

C (Comparison) = any intervention or non-treatment control

O (Outcome) = PA measure (device based, participant report)

S (Study) = randomised controlled trials

### Study selection

Two reviewers will independently screen the titles and abstracts, and then papers that fulfil the eligibility requirements will be read in full and their suitability for inclusion will be independently decided, with disagreements resolved by a third author, if required. Results will be transferred into the Covidence software ( https://www.covidence.org) ^51^, for title/abstract screening.

### Data extraction and quality assessment

Study details and data will be extracted using a customised form. Theoretical information and intervention details including approaches, dose etc., will be extracted. Qualitative data will be extracted to understand people's experiences of receiving the intervention and barriers and enablers to consider in our future intervention. Risk of bias will be assessed independently by two reviewers with disagreements resolved by a third author, if required. Risk of bias will be used to weight the evidence in a subsequent meta-analysis.

### Data synthesis and analysis

Where appropriate a meta-analysis (quantitative data) or meta-synthesis (qualitative data) will be conducted. The meta-analysis will investigate the effectiveness of non-digital interventions for PA maintenance in people with arthritis. Where such synthesis is not possible, a narrative synthesis will be conducted.

## Phase 2: Observation and qualitative study

Observational Analysis: Objective (ii) Identify facilitators/deficits in supervised exercise classes to maintain PA.

### Methods

We will conduct an observational analysis of a physiotherapy led, structured exercise programme for those living with arthritis, to better understand what behaviour change components are being used that might support PA maintenance. This will be done in three steps.

1) We know that physiotherapists will be trained in advance of delivering the exercise programmes. We will review the training materials for behaviour change components related to PA.
2) We will then map these behaviour change components to The Behaviour Change Technique Taxonomy (BCTT v1) ^52^.
3) We will then undertake a direct face-to-face observational analysis of the delivery of the exercise classes, to explore which BCTs are used to support PA maintenance. A detailed checklist (informed by the evidence synthesis in part one and the review of the training materials) will be completed during each session by a member of the research team. The classes will also be audio-record classes to allow for further post observation analysis of the behavioural components for example the communication style and behaviour change techniques delivered by the class leader. Any supporting materials used by the delivering physiotherapist during the classes will also be reviewed for presence of BCTs.

### Analysis

Steps 1 and 2: The BCTT v1, an extensive hierarchically organised taxonomy of 93 distinct behaviour change techniques (BCTs), will be used to identify the core BCTs recommended in the programme training materials. We will distinguish between those BCTs used for supporting in-class exercise versus those supporting day-to-day PA maintenance, as informed by phase 1 results. This will be done by reviewing the training material for the presence of core BCTs linked to PA in-class and PA maintenance i.e. retrofitting the BCTs to the material provided in the manuals. In the event of uncertainties or disagreements with the BCT coding, consensus will be achieved through discussion amongst the wider research team. 

Step 3:

Analysis: The content of the checklists and the audio recordings will be analysed for the presence of BCTs (as informed by steps 1 and 2 above) to establish differences between expected BCT delivery and actual BCT delivery. This method has been adapted from Hawkes *et al*. ^53^ (Hawkes paper).

### Qualitative study

Objective (iii) Identify the support needs of people living with arthritis, their family members/friends and the delivering physiotherapists, to maintain PA.

### Methods

This study phase will be conducted in accordance with the Consolidated criteria for Reporting Qualitative research (COREQ) ^54^. Focus groups/interviews will be held for with those living with arthritis who have attended a physiotherapy led, structured exercise programme, their family/friends and the delivering therapists. The aim is to identify barriers, facilitators and strategies of PA maintenance in those living with arthritis.

The content of the topic guides will be shaped by the clinical and academic experience of the research team based on the Theoretical Domains Framework (TDF) ^55^. Using the TDF domains to structure the topic guide ensures a robust theoretical basis to the questioning that covers a wide range of behavioural influences ^56^. The topic guide will be piloted with PPI co-researchers in advance of the first focus group and amended as required. This data will not be included in analysis. The topic guides will be refined on an iterative basis for each focus group and are not intended to be prescriptive.

### Recruitment

We will recruit people with arthritis who have attended a physiotherapy led structured exercise programme for those living with arthritis, their family members/friends and the delivering physiotherapists We will consider the diversity of people with arthritis in terms of age, gender, ethnicity, geographical location, severity of arthritis, socio-economic status, digital literacy and any other factors identified as important by our PPI panel.

### Conduct of the focus groups

Three to six focus groups are likely to identify 90% of themes within a discussion, with a target number per group (5–8), based upon previous recommendations ^57^. Therefore at least six focus groups will be held with people living with arthritis, and separately with family members/friends and physiotherapists (n=3). The focus groups will be held either online or in-person depending on peoples' preferences. In the instance where a focus group is not possible, individual interviews will be considered. It is anticipated that each discussion will last around 45–60 minutes. 

### Data analysis

Verbatim audio recordings will be transcribed, pseudonymized, and imported into NVivo software ^58^. Data will be analysed using the Framework Method which incorporates both deductive and inductive data analysis ^59^. This method accommodates our predefined topics based on the TDF domains while remaining open to the emergence of additional inductive themes. The TDF will provide a comprehensive framework for assessing the behaviour and a method for informing the design of an appropriately targeted intervention ^55^.

At least one member of the research team will read and independently code all transcripts using the domains of the TDF. Another researcher will verify all codes and meet to compare and discuss coding decisions. Where coding disagreement occurs, areas of difference will be resolved through discussion and review of original transcripts. If key elements of the data cannot be adequately described or captured by the TDF constructs, inductive coding may be used. This approach will allow for the inclusion of both a priori (e.g. TDF domains) and other emergent factors. Preliminary results and interpretations will be shared with the wider research group including those living with arthritis.

## Phase 3: Finalise intervention prototype: Objective (iv) Co-develop a PA maintenance intervention prototype

### Methods

In this study, we use the “Guidance for reporting intervention development studies in health research” checklist to detail the development process ^37^. The results from previous phases (1 and 2) will be triangulated by the research team. From this, we will compile options with respect to a range of behaviour change techniques that could/should be included in a future intervention, and approaches to intervention delivery. This will allow for the generation of a draft outline of potential intervention options to allow those living with arthritis maintain their PA levels long-term. The APEASE criteria (Acceptability, Practicability, Effectiveness and cost-effectiveness, Affordability, Safety/side-effects, Equity) will be used when making decisions about which intervention would be most appropriate ^60^.

It is important that in this proposed phase, we include people living with arthritis and other key stakeholders to participate in decision making around the content and delivery of the intervention. Participatory workshops can provide an efficient method of bringing key stakeholders together to stimulate discussion on the proposed options and to seek consensus on the design, content and mode of delivery of an intervention. The workshops will use an adapted version of nominal group techniques to make decisions, which is a less time consuming and more cost-effective approach in comparison to other consensus generating methods (e.g., Delphi) ^61, 62^. It is hoped that by engaging these people in making decisions, it will lead to a greater buy-in and increase the chance of successful implementation. Involving them is well recognised as a valuable way to ensure their expectations, needs and preferences are met in a meaningful way ^63^. The findings from the participatory workshops will influence the design of the final intervention protocols, which will be feasibility tested in future research (phase 4 of this project).

### Recruitment

We will complete two separate workshops, with people living with arthritis (n=20–40) and stakeholders (n=20–40). Stakeholders will include family members of those living with arthritis, health care professionals, charitable organisations, governmental agencies. This list is not exhaustive and will include any persons or groups who are working/volunteering or providing support in or the area of arthritis care and/or PA promotion.

### Conduct of the workshops

The workshops will be held as one-off events and will adopt a consultation style approach. The workshops will be facilitated by a trained moderator. The workshops will ideally be held at an in-person event at a day, time and place to suit the majority of participants. In the event that an in-person event is not possible, an online option will be offered. The methods employed for both workshops will be broadly similar. Basic demographic data will be collected including questions around age category and gender. 

The exact schedule and content of the workshop will be informed by the previous phases of this wider project. At the outset of each workshop, the attendees will be presented with a brief summary of the research completed to date, background to the research and summary findings proposed interventions/solutions, prepared by the research team. The workshop will be structured into small group discussions at separate tables with a member of the research team acting as a facilitator/note taker in order to summarise the qualitative feedback. The moderator will introduce a topic and instruct the group on the time allowed for discussions. A series of pre-determined summary statements will be presented to participants relating to the topics such as delivery mode, content, and frequency of delivery, how the intervention is proposed to fit in its wider context and the relevance for people living with arthritis. Participants will then be asked to vote on or rank the solutions or options posed using an online polling software such as Mentimeter ( https://www.mentimeter.com) ^64^. The facilitators will de-brief post event and collate any qualitative outputs that were generated. Consensus statements will be compiled that reflect the groups’ views regarding the proposed intervention to be feasibility tested in Phase 4.

### Data analysis

Both quantitative and qualitative outputs will be generated during the workshops. Analysis and synthesis of the results will be carried out during the events and afterwards. Qualitative data will be generated through discussions, comments provided by participants throughout the day and open-ended answers on the online polling software. Quantitative outputs will be generated through the process of voting and ranking. Whilst some of this analysis will occur ‘live’ throughout the day, further exploration of the metrics will be conducted post event. Results from the voting and ranking process, and demographic data will be explored using simple descriptive statistics. The results will provide a set of prioritised solutions and recommendations that represent the groups’ preferences. In the event that ambiguity remains, or where views from each workshop strongly oppose one another, we will hold follow up meetings with the wider research team and key stakeholders to reach final agreement on these issues.

## Phase 4: Feasibility study. Objective (v) Test the feasibility of the PA maintenance intervention prototype

The fourth and final phase of the project will test the feasibility of the newly developed multicomponent PA maintenance intervention for those living with arthritis after exit from a structured exercise programme. 

The objectives are to:

- determine acceptability in terms of the incidence of adverse events and level of overall satisfaction in those living with arthritis
- explore sensitivity to change of PA maintenance measures
- identify adherence to the intervention
- investigate differences in recruitment depending on the recruitment route and factors explaining these differences
- explore the acceptability of outcome measures to be used in a future controlled trial

### Methods

In this mixed-methods study, we will use a pre-post evaluation design to test intervention feasibility. The reporting of this will follow the an adapted version of the Consolidated Standards of Reporting Trials (CONSORT) 2010 statement with extension to randomised pilot and feasibility trials ^65^ and the Template for Intervention Description and Replication (TIDieR) checklist ^66^. This trial will be registered with clinicaltrials.gov.

### Study population

Adults living with arthritis who have completed a physiotherapy led structured exercise programme in Ireland will be eligible to participate.

### Recruitment

This trial aims to recruit 30 participants. This is in line with recommendations where it is suggested that a sample size of n=30 is appropriate to answer the questions posed by a feasibility trial ^67, 68^. This will also allow for exploration of different recruitment experiences from up to three recruitment streams. Recruitment to the study will be via existing structured exercise programmes for people living with arthritis in Ireland.

### Intervention

The intervention modality, content and duration will be based upon the decisions made by the workshops in phase 3. The intervention will focus on supporting maintenance of PA in a way that is relevant to each individual through tailoring to address differences in physical capacity. Given the likely importance of self-regulatory processes for maintaining a behaviour, the intervention will likely include components to support the individual to consider their own perceived barriers and facilitators to maintaining PA in the longer term at an individual, organisational, structural and system level. In order to support people with arthritis to be active independently following a supervised exercise class communication approaches that empower participants to be autonomous, as recommended by the World Health Organisation (WHO) in their primary care toolkit ^69^, will be considered. Such approaches, including the 5As and motivational interviewing, have already been tested for initiation of PA by our team ^70– 72^.

### Outcomes

***Interviews***

Qualitative data will be the main mechanism used to understand the potential of the intervention to effect PA maintenance in people with arthritis. Individual interviews will be conducted with participants with arthritis to allow participants share experiences of the delivery of the intervention and barriers to implementation. These will be done both before and after the intervention to explore the participants’ needs and expectations around PA maintenance, and how/if these were met.

***Other outcomes***

Given our focus on intervention development, we will not measure clinical end points. We will consider process outcome measures instead based on our logic model developed in phase 1. The exact process outcomes to be measured will be decided by the researchers and PPI members, as results from Phases 1–3 become available.

### Data analysis

The main focus of our analysis will be on the qualitative data to understand peoples’ experiences of the intervention, their satisfaction and acceptability of the intervention and any improvements needed. This data will be transcribed verbatim, and interpretation, synthesis and data reduction will be undertaken to identify relevant themes as per Thematic Analysis approach ^73^. Qualitative findings will be used alongside quantitative outcomes related to process evaluation to understand how the intervention was experienced by participants and any changes that they think would improve the intervention. 

We will collect demographic and clinical characteristic data. As this is a feasibility study, significance tests on any quantitative data will not be performed. Descriptive analyses will be used, including means, standard deviations, medians, inter-quartile range (IQR), frequencies, proportions and 95% confidence intervals where appropriate.

## Conclusion

The article describes the protocol for a multi-phase programme of work which aims to co-develop and feasibility test a PA maintenance intervention for those living with arthritis, after exit from a structured exercise programme. There is a clear need for participatory research in developing interventions to ensure diverse patient experiences and perspectives are heard and inform programme design. This overall study offers a novel approach, based on robust behaviour change principles, informed by the published literature, stakeholder input and PPI involvement to achieving a more active lifestyle in the long-term for people living with arthritis.

## Ethics and consent

Written informed consent will be obtained from participants as applicable. Ethics approval will be sought through the RCSI Human Research Ethics Committee and other ethics committees as required.

## Data Availability

No data are associated with this article.
