# Supplementary material for: MAintAin Activity in Arthritis: A study protocol of the co-development and feasibility testing of a physical activity maintenance intervention
Source: HRB Open Res. 2025 Apr 28;7:75. Originally published 2024 Dec 10. [Version 2] doi: 10.12688/hrbopenres.14002.2 (PMC12082073; doi:10.12688/hrbopenres.14002.2)
Supplement: Peer review history

# Reviewer report: Reviewer response for version 2

**DOI:** 10.21956/hrbopenres.15544.r47045

**Published:** 2025-05-14

**Authors:** Rebecca Grainger

**Affiliations:** 1 Department of Medicine, University of Otago, Dunedin, Otago, New Zealand

I have no further comments to make

Is the study design appropriate for the research question?

Yes

Is the rationale for, and objectives of, the study clearly described?

Partly

Are sufficient details of the methods provided to allow replication by others?

Yes

Are the datasets clearly presented in a useable and accessible format?

Yes

Reviewer Expertise:

Arthritis, technology, participatory interventions

I confirm that I have read this submission and believe that I have an appropriate level of expertise to confirm that it is of an acceptable scientific standard.

# Reviewer report: Reviewer response for version 2

**DOI:** 10.21956/hrbopenres.15544.r47044

**Published:** 2025-05-09

**Authors:** Kristin Houghton

**Affiliations:** 1 Pediatric Rheumatologist BC Children’s Hospital, Vancouver, Canada

The authors have addressed reviewer comments.  I have no further comments to make.

Is the study design appropriate for the research question?

Yes

Is the rationale for, and objectives of, the study clearly described?

Yes

Are sufficient details of the methods provided to allow replication by others?

Yes

Are the datasets clearly presented in a useable and accessible format?

Not applicable

Reviewer Expertise:

Physical activity, arthritis

I confirm that I have read this submission and believe that I have an appropriate level of expertise to confirm that it is of an acceptable scientific standard.

# Reviewer report: Reviewer response for version 1

**DOI:** 10.21956/hrbopenres.15368.r44519

**Published:** 2025-01-11

**Authors:** Rebecca Grainger

**Affiliations:** 1 Department of Medicine, University of Otago, Dunedin, Otago, New Zealand

This protocol describes a four-phase research plan with the aim to develop and pilot a physical activity maintenance intervention for people living with arthritis, which will be implemented after a physiotherapist-led structured exercise programme.  

 The protocol is clearly written and easy to follow.  The details provided suggest to me that the team has all the knowledge and expertise to design high-quality research.  

 The co-design in phase 4 is commendable and a strength of the protocol.

 I have a few comments for reflection by the authors:

 1.The study focuses on maintaining physical activity (PA) after a physiotherapy (PT) intervention. It has therefore been assumed that this intervention is effective in increasing PA and indeed is the most effective and efficient method of doing so.  The protocol could have been strengthened by addressing this. It is somewhat reassuring to see the Phase 2 of the study which is really to improve the current exercise classes. 

 2. In light of the above comment, I was somewhat surprised that Phase 1 of the study had not been completed before the remainder of the study was designed.  The authors may wish to consider a contingency plan, in case data suggest an intervention other than supervised exercise programme may have better evidence for effectiveness (or less good evidence but better cost-effectiveness or easier to scale etc).  It is also worth clarifying how the recurrent PT exercise programme will be adapted to enhance behavioural change and incorporate evidence -based BC techniques to enhance PA maintenance.  Will future PT exercise classes be different?

 3. As a related point, what is known about the fidelity of current PT exercise classes - how codified are these, and how much variation is there?  This may threaten validity of phase 2 data and, unless addressed, could influence the ability for the programme to be replicated. 

 I wish the authors all the best with this interesting work and look forward to seeing the outcomes.

Is the study design appropriate for the research question?

Yes

Is the rationale for, and objectives of, the study clearly described?

Partly

Are sufficient details of the methods provided to allow replication by others?

Yes

Are the datasets clearly presented in a useable and accessible format?

Yes

Reviewer Expertise:

Arthritis, technology, participatory interventions

I confirm that I have read this submission and believe that I have an appropriate level of expertise to confirm that it is of an acceptable scientific standard, however I have significant reservations, as outlined above.

# Reviewer report: Reviewer response for version 1

**DOI:** 10.21956/hrbopenres.15368.r44260

**Published:** 2025-01-02

**Authors:** Kristin Houghton

**Affiliations:** 1 Pediatric Rheumatologist BC Children’s Hospital, Vancouver, Canada

The authors propose a multi-staged study protocol on physical activity (PA) in people living with arthritis, focusing on maintaining PA after completion of a structured exercise program. The study protocol includes an initial review the literature (evidence synthesis), followed by observation of current practice (observational qualitative study). The evidence  acquired in these first stages will be used to inform and finalize an intervention (intervention prototype). The final stage is a feasibility study using pre-post, mixed methods.  

 Strengths

 Participatory research - Patient partner involvement, key stakeholders identified for participation 

 Focus on behavioural change principles

 Weaknesses

 Small N for the amount of work put into this protocol (note that N=30 is well justified in the manuscript)

 Feasibility for larger scale implementation 

 Suggestions

 Consider metrics for psychological and mental health support and whether peer support is a viable option.

Is the study design appropriate for the research question?

Yes

Is the rationale for, and objectives of, the study clearly described?

Yes

Are sufficient details of the methods provided to allow replication by others?

Yes

Are the datasets clearly presented in a useable and accessible format?

Not applicable

Reviewer Expertise:

Physical activity, arthritis

I confirm that I have read this submission and believe that I have an appropriate level of expertise to confirm that it is of an acceptable scientific standard, however I have significant reservations, as outlined above.

# Response

**Published:** 2025-04-15

**Authors:** Aoife Stephenson

**Affiliations:** School of Physiotherapy, Royal College of Surgeons in Ireland, Dublin, Leinster, Ireland

Thank you so much for your time in reviewing our work.

 In response to your specific queries please see below. 

**Comment: Weaknesses: Small n for the amount of work put into this protocol (note that n=30 is well justified in the manuscript)**

**Feasibility for larger scale implementation**

**Response: **thanks for your feedback, we are following the MRC (ref 34) and Index guidance (refs 35-37) to carefully develop our complex intervention prior to initial testing. We therefore had to balance how much testing we could include against the time needed to carefully develop the intervention prior to testing (phases 1-3) in this 2.5 year project.  We anticipate that further clinical testing can be carried out in a future funded feasibility randomised controlled trial. 

**Comment: Suggestions: Consider metrics for psychological and mental health support and whether peer support is a viable option.**

**Response: **Thanks for comment. This will be taken into consideration, particularly if deemed important in focus groups (phase 2) and workshops (phase 3).

# Response

**Published:** 2025-04-15

**Authors:** Aoife Stephenson

**Affiliations:** School of Physiotherapy, Royal College of Surgeons in Ireland, Dublin, Leinster, Ireland

Reviewer 2: Thank you so much for your time in reviewing our work.

 In response to your specific queries please see below. 

**Comment: The study focuses on maintaining physical activity (pa) after a physiotherapy (pt) intervention. It has therefore been assumed that this intervention is effective in increasing pa and indeed is the most effective and efficient method of doing so.  The protocol could have been strengthened by addressing this. It is somewhat reassuring to see the phase 2 of the study which is really to improve the current exercise classes.**

**Response:** Thanks for this comment, and we agree that the work we are doing in phase 2 of the study will help us to identify behaviour change techniques being used already, and those that could be added to improve the exercise classes.

**Comment: In light of the above comment, i was somewhat surprised that phase 1 of the study had not been completed before the remainder of the study was designed.  The authors may wish to consider a contingency plan, in case data suggest an intervention other than supervised exercise programme may have better evidence for effectiveness (or less good evidence but better cost-effectiveness or easier to scale etc).  It is also worth clarifying how the recurrent pt exercise programme will be adapted to enhance behavioural change and incorporate evidence -based bc techniques to enhance pa maintenance.  Will future pt exercise classes be different?**

****

**Response: **Thanks for this important comment.  Prior to starting this study, we had completed a scoping review, a systematic review was underway to inform phase 1 so we had a good sense of the literature already.  Pragmatically, supervised exercise classes are commonly recommended in clinical guidelines for the management of a range of clinical conditions including arthritis, and there is evidence of short-term effects in activating people.  The work we have done in phase 2 will be disseminated to influence the delivery of these classes; however, it is not within the scope of the current project to change the existing exercise classes.  We agree that this will be important to consider in future studies. 

**Comment: As a related point, what is known about the fidelity of current pt exercise classes - how codified are these, and how much variation is there?  This may threaten validity of phase 2 data and, unless addressed, could influence the ability for the programme to be replicated.**

****

**Response:** Thanks for your helpful comment, and we agree that fidelity of current classes is important.  The physiotherapists delivering the classes will all been trained in the delivery and this is a strength.  We will have a snapshot of fidelity from phase 2 of our project. Our aim will be to recruit from those classes where it was delivered as intended according to the training.
